# Cardiac Electrophysiological Effects of the Sodium Channel-Blocking Antiepileptic Drugs Lamotrigine and Lacosamide

**DOI:** 10.3390/ph18050726

**Published:** 2025-05-15

**Authors:** Julian Wolfes, Philipp Achenbach, Felix K. Wegner, Benjamin Rath, Lars Eckardt, Gerrit Frommeyer, Christian Ellermann

**Affiliations:** Department of Cardiology II (Electrophysiology), University Hospital Münster, Albert-Schweitzer-Campus 1, 48149 Münster, Germany

**Keywords:** Langendorff, sotalol, long-QT syndrome, arrhythmia, sudden cardiac death, sodium channel block, seizure suppressants

## Abstract

**Background:** The two antiepileptic drugs lacosamide and lamotrigine exert their antiepileptic effect by inhibiting sodium channels. Lacosamide enhances the inactivation of sodium channels, while lamotrigine inhibits the activation of the channel. Interactions with sodium channels also play an interesting role in cardiac pro- and antiarrhythmia, with inhibition of inactivation, in particular, being regarded as potentially proarrhythmic. Therefore, the ventricular electrophysiologic effects of lacosamide and lamotrigine were investigated in an established experimental whole-heart model. **Methods:** A total of 67 rabbit hearts were allocated to four groups. Retrograde aortic perfusion was performed using the Langendorff setup. The action potential duration at 90% repolarization (APD_90_), QT intervals, spatial dispersion of repolarization, effective refractory period, post-repolarization refractoriness, and VT incidence were determined. The electrophysiological effects of lacosamide and lamotrigine were investigated in increasing concentrations on the natively perfused heart. On the other hand, perfusion with the I_Kr_-blocker sotalol was performed to increase arrhythmia susceptibility, followed by perfusion with lacosamide or lamotrigine to investigate the effects of both in a setting of increased arrhythmia susceptibility. Perfusion with lacosamide and lamotrigine tended to decrease APD_90_ and QT-interval. As expected, perfusion with sotalol led to a significant increase in APD_90_, QT interval, and arrhythmia incidence. Additive perfusion with lacosamide led to a further increase in arrhythmia incidence, while additive perfusion with lamotrigine led to a decrease in VT incidence. **Conclusions:** In this model, lacosamide showed proarrhythmic effects, especially in the setting of an additive prolonged QT interval. Lamotrigine showed no significant proarrhythmia under baseline conditions and rather antiarrhythmic effects with additive QT prolongation.

## 1. Introduction

Sudden unexpected death in epilepsy (SUDEP) is a major cause of death in epilepsy patients [1]. In addition to hypoxia in the course of persistent tonic-clonic convulsions [2], cardiac arrhythmias [3] are also often suspected as possible causes of sudden death. It is unclear to what extent this is due to genetic predispositions, which can be detected more frequently in SUDEP patients [4], or to possible proarrhythmic factors of epilepsy medication. Lacosamide and lamotrigine are two antiepileptic drugs whose mechanism of action is based on the inhibition of sodium channels. While lacosamide is used to treat focal seizures with or without secondary generalization, lamotrigine is used not only to treat focal or generalized seizures but also as a mood stabilizer to prevent depressive episodes in patients with bipolar disorder. Various groups and a meta-analysis indicated ECG changes and rhythm events with lacosamide [5,6,7], which led to an FDA warning [5]. With lamotrigine, these effects appear to be described less frequently overall [8], although ECG changes are also described in individual cases, particularly with Brugada-like ECG changes [9,10,11]. Interestingly, both preparations show a different interaction with sodium channels, with lacosamide primarily enhancing the inactivation of sodium channels [12], while lamotrigine blocks the ability to activate sodium channels [13]. Due to the divergent proarrhythmia risk of both drugs described in the literature, the correlation of SUDEP patients with proarrhythmic gene mutations, and the described divergent interaction with sodium channels, we wanted to characterize both substances in our Langendorff model of the isolated rabbit heart.

## 2. Results

### 2.1. Lacosamide

**Figure 1:**
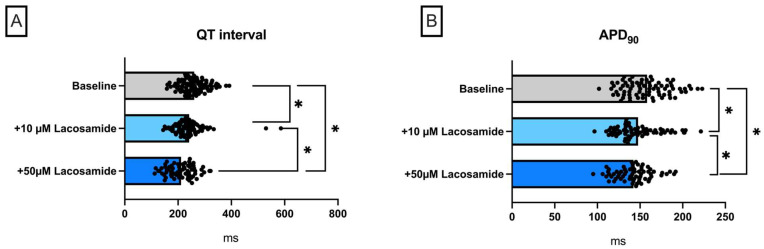

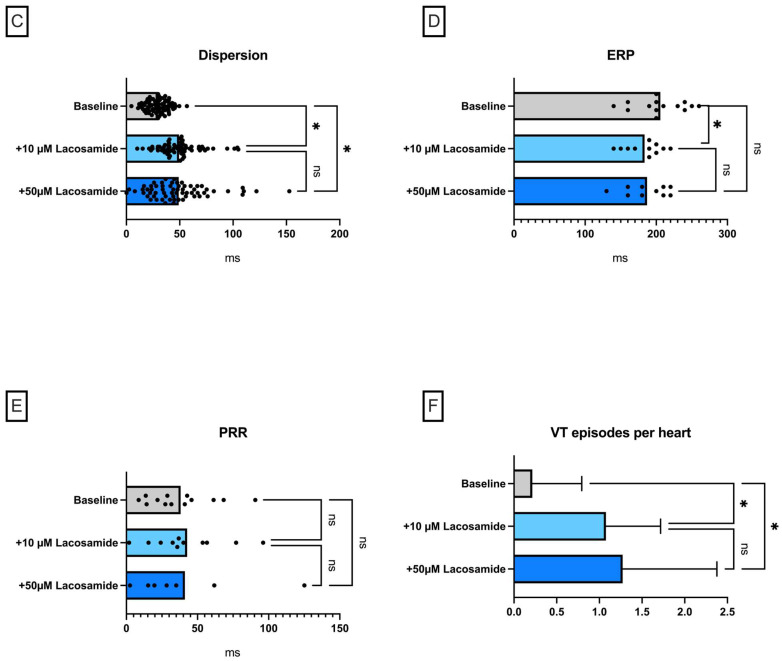
(**A**) Overall QT interval and (**B**) APD_90_. Concentration-dependent effect of lacosamide on (**C**) spatial dispersion of repolarization, (**D**) effective refractory period (ERP), (**E**) post-repolarization refractoriness (PRR), and (**F**) number of ventricular tachycardia (VT)/fibrillation (VF) episodes (* = *p* < 0.05). The data derived from 14 hearts (n = 14) and were analyzed employing a mixed-effects model. ns = non-significant.

Perfusion with lacosamide (Figure 1) caused a significant shortening of QT interval (baseline: 261 ± 36 ms; +10 µM lacosamide: 240 ± 45 ms (*p* < 0.05); +50 µM lacosamide: 210 ± 48 ms (*p* < 0.05)) and APD_90_ (baseline: 158 ± 23 ms; +10 µM lacosamide: 147 ± 18 ms (*p* < 0.05); +50 µM lacosamide: 142 ± 16 ms (*p* < 0.05)) (Figure 1). This was accompanied by a significant increase in spatial dispersion. The effective refractory period and PRR were not significantly changed. The incidence of spontaneous ventricular arrhythmia episodes was tendentially increased (baseline: 0.21 ± 0.58 episodes; +10 µM lacosamide: 1.08 ± 0.64 episodes (*p* < 0.05); +50 µM lacosamide: 1.27 ± 1.1 episodes (*p* < 0.05)).

#### Lacosamide in a Model of QT Prolongation

**Figure 2:**
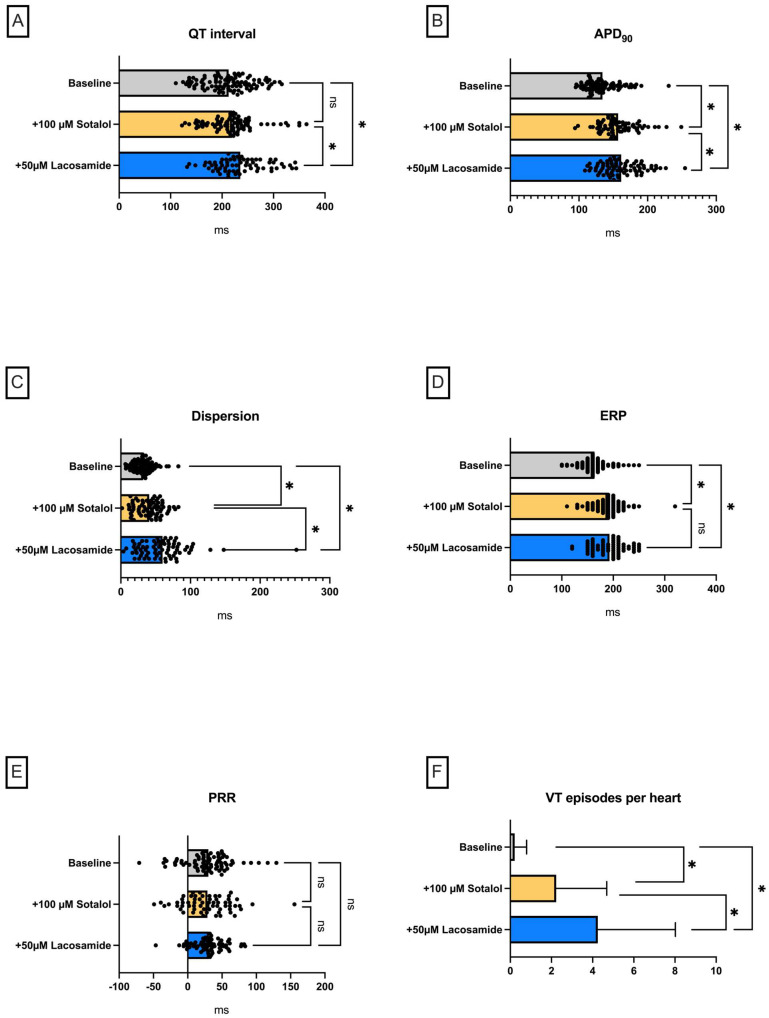
(**A**) Overall QT interval and (**B**) APD_90_. Effect on (**C**) spatial dispersion of repolarization, (**D**) effective refractory period (ERP), (**E**) post-repolarization refractoriness (PRR), and (**F**) number of ventricular tachycardia (VT)/fibrillation (VF) episodes (* = *p* < 0.05). The data derived from 15 hearts (n = 15) and were analyzed employing a mixed-effects model. ns = non-significant.

Perfusion with the I_Kr_-blocker sotalol (Figure 2) was used to induce drug-induced LQT-2 syndrome. As expected, perfusion with sotalol led to a significant prolongation of APD_90_ (baseline: 134 ± 21 ms; +100 µM sotalol: 157 ± 22 ms (*p* < 0.05)) but did not significantly prolong the QT interval (baseline: 213 ± 45 ms; +100 µM sotalol: 219 ± 46 ms (*p* = ns)) (Figure 2) Additive perfusion with lacosamide here led to further slight prolongation of APD_90_ (+50 µM lacosamide: 161 ± 24 ms (*p* < 0.05)) and QT interval (+50 µM lacosamide: 234 ± 45 ms (*p* < 0.05)). This was accompanied by a significant increase in spatial dispersion. There was also a significant increase in ERP with no change in PRR due to prolonged APD_90_. Under perfusion with sotalol, there was a significant increase in VT incidence, whereby the additive lacosamide perfusion caused a further increase in VT incidence (baseline: 0.21 ± 0.58 episodes; +100 µM sotalol: 2.23 + 2.45 episodes (*p* < 0.05); +50 µM lacosamide: 4.25 ± 3.77 episodes (*p* < 0.05)).

### 2.2. Lamotrigine

**Figure 3:**
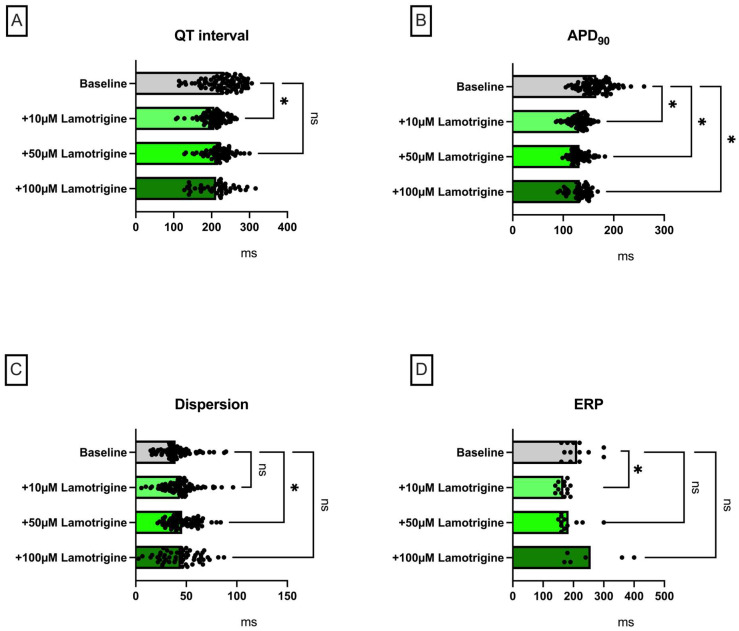

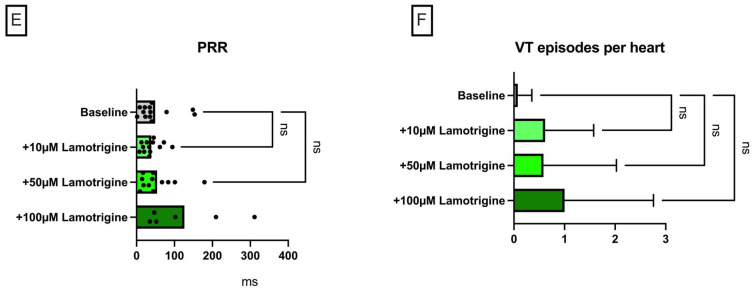
(**A**) Overall QT interval and (**B**) APD_90_. Concentration-dependent effect of lamotrigine on (**C**) spatial dispersion of repolarization, (**D**) effective refractory (ERP), (**E**) post-repolarization refractoriness (PRR) and (**F**) number of ventricular tachycardia (VT)/fibrillation (VF) episodes (* = *p* < 0.05)**.** The data derived from 13 hearts (n = 13) and were analyzed using a mixed-effects model. ns = non-significant.

Lamotrigine perfusion led to a significant shortening of APD_90_ (baseline: 166 ± 24 ms; +10 µM lamotrigine: 132 ± 13 ms (*p* < 0.05); +50 µM lamotrigine: 132 ± 14 ms (*p* < 0.05); +100 µM lamotrigine: 131 ± 19 ms (*p* < 0. 05)) (Figure 3), whereby this effect was not equally significant for QT interval (baseline: 231 ± 38 ms; +10 µM lamotrigine: 208 ± 27 ms (*p* < 0.05); +50 µM lamotrigine: 216 ± 34 ms (*p* = ns); +100 µM lamotrigine: 210 ± 42 ms (*p* = ns)). Dispersion tended to be significantly increased under perfusion with lamotrigine, albeit not at all concentrations. Under low concentrations of lamotrigine, there was a decrease in ERP, whereas under the maximum lamotrigine concentrations, no significant increase in ERP was observed. The PRR behaved in a similar way, whereby a pronounced scattering of the values resulted in the fact that the tendency of the PRR to increase under maximum doses did not reach the threshold value of statistical significance. The incidence of ventricular arrhythmia episodes was not significantly increased.

#### Lamotrigine in a Model of QT Prolongation

**Figure 4:**
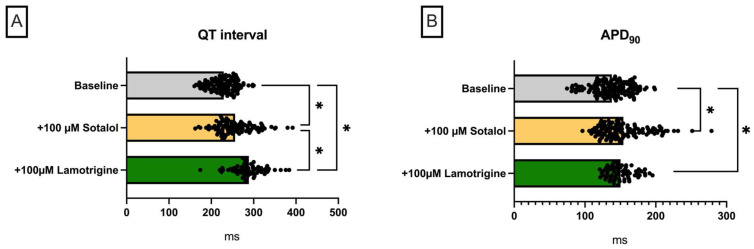

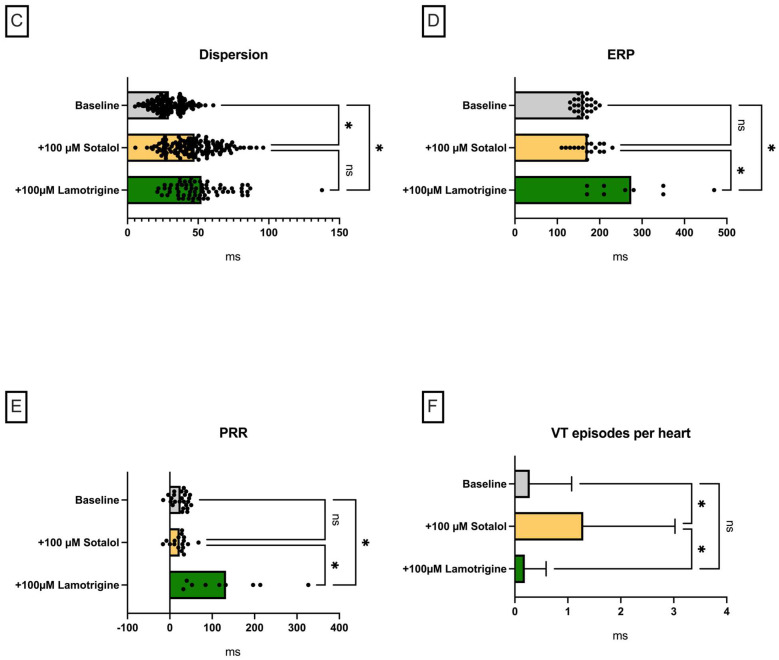
(**A**) Overall QT interval and (**B**) APD_90_. Effect on (**C**) spatial dispersion of repolarization, (**D**) effective refractory period (ERP), (**E**) post-repolarization refractoriness (PRR), and (**F**) number of ventricular tachycardia (VT)/fibrillation (VF) episodes (* = *p* < 0.05). The data derived from 25 hearts (n = 25) and were analyzed using a mixed-effects model. ns = non-significant.

Predictably, perfusion with sotalol also led to a significant prolongation of QT interval in this group (baseline: 229 ± 30 ms; +100 µM sotalol: 256 ± 44 ms (*p* < 0.05)) and APD_90_ (baseline: 138 ± 26 ms; +100 µM sotalol: 154 ± 32 ms (*p* < 0.05)) (Figure 4). Additive perfusion with lamotrigine in this setting led to a further prolongation of QT interval (+100 µM lamotrigine: 289 ± 37 ms (*p* < 0.05)) with no significant effect on APD_90_ (+100 µM lamotrigine: 150 ± 18 ms (*p* = ns)). Dispersion was significantly prolonged under sotalol, as also expected, while additive perfusion with lacosamide had no effect in this regard. The ERP was significantly prolonged under additive lacosamide perfusion. Consequently, PRR was also significantly prolonged with lacosamide. The incidence of ventricular arrhythmia episodes increased significantly under perfusion with sotalol and was reduced below the baseline level by additive perfusion with lacosamide.

## 3. Discussion

The Langendorff study on the electrophysiological effects of lacosamide and lamotrigine in an established whole-heart model showed the following main findings.

- (I) Both substances led to a trend towards a decrease in action potential duration and/or QT interval under baseline conditions.
- (II) Perfusion with both sodium channel blockers did not lead to a significant increase in arrhythmia incidence under baseline conditions.
- (III) In the course of a drug-induced LQT syndrome, perfusion with lacosamide led to a significant increase in QT interval and arrhythmia incidence.
- (IV) In the course of a drug-induced LQT syndrome, perfusion with lamotrigine did not lead to a significant increase in QT interval. However, the incidence of arrhythmia was significantly reduced. This observation was accompanied by an increase in PRR.

### 3.1. Sodium Channel Inactivation

To better understand the mechanisms behind lamotrigine’s and lacosamide’s actions, it is important to consider the two distinct mechanisms that underlie fast and slow sodium channel inactivation. Inactivation is an inherent characteristic of NaV channels that regulates cellular excitability by controlling the availability of the channel. Fast inactivation of voltage-gated sodium channels is crucial for the efficient generation and propagation of action potentials. Slow inactivation plays a crucial role in regulating membrane excitability, firing properties, and spike frequency adaptation. Impaired slow inactivation is linked to various cell excitability disorders, such as long-QT syndrome and idiopathic ventricular fibrillation [14]. Fast inactivation occurs when the channel’s inner gate (located in the sixth transmembrane helix, S6) is pinched upon the binding of the inactivation particle (a short cytoplasmic loop between domains III and IV [15], containing the critical and indispensable [16] Ile-Phe-Met (IFM) motif [17] to a lateral binding pocket [18], as demonstrated in recent phototrapping experiments [19,20]. In contrast, slow inactivation results from conformational changes in the selectivity filter region and the outer S6 helices [21], a process known as C-type inactivation [22]. Slow and fast inactivation are both voltage-dependent mechanisms. Of note, mutations that impact fast and slow inactivation seem to interact, even though they are located at distant positions within the channel [22]. It is worth noting that lacosamide and lamotrigine bind to a common, shallow pocket beneath the intracellular gate (called site BIG), though they interact in different ways. Additionally, both compounds occupy a second position within the central cavity (site C), as revealed by molecular docking studies using 3D models of NaV channels [23,24,25].

### 3.2. Simulation of a Model of Reduced Repolarization Reserve

In two of the experimental groups, sotalol was administered to facilitate the occurrence of ventricular arrhythmias by reducing the repolarization reserve [26]. As an I_Kr_ inhibitor, sotalol, with its well-characterized torsadogenic properties, serves as a representative agent with proarrhythmic potential. Consequently, sotalol administration resulted in prolonged cardiac repolarization, increased spatial dispersion of repolarization, and the occurrence of ventricular tachycardia. It is primarily the increased spatial dispersion of repolarization, rather than the sole prolongation of cardiac repolarization, that contributes to the arrhythmic risk associated with sotalol [27]. Notably, it is challenging to induce arrhythmias in hearts with preserved repolarization reserve. Therefore, reducing the repolarization reserve (e.g., through I_Kr_ inhibition) lowers the threshold for drug-induced arrhythmias, thereby increasing the sensitivity of the present model [28]. It is important to emphasize that I_Kr_ inhibition plays a crucial role in drug-induced proarrhythmia [29] and is a key factor in contemporary drug safety testing strategies, as outlined in guidelines by the International Conference on Harmonisation of Technical Requirements for Registration of Pharmaceuticals for Human Use (ICH) [30]. In addition, QT interval prolongation or APD prolongation may be seen in the course of heart failure, with reduced I_Kr_ kinetics [31].

### 3.3. Electrophysiological Effects of Lacosamide

Perfusion with lacosamide led to a significant decrease in APD_90_ and QT interval. The incidence of VT was not significantly increased. The ERP and PRR were not significantly prolonged. To better understand the underlying mechanisms, it is essential to consider the different potential antiepileptic actions. Inactivation of sodium channels is crucial for the effects of most antiepileptic drugs. While many antiepileptic drugs target both fast and slow inactivation processes, lacosamide is unique in that it selectively enhances slow inactivation of voltage-gated sodium channels without affecting fast inactivation gating [32].

It is important to note that slow inactivation of sodium channels contributes to the late sodium current (I_Na,L_) [33], which is active during the plateau phase and thus affects action potential morphology. An upregulated I_Na,L_ impairs repolarization and increases intracellular sodium concentration in cardiomyocytes, leading to cardiac arrhythmias [34]. Therefore, the observed shortening of the action potential duration can be explained by the enhancement of slow sodium channel inactivation [12]. Inhibition of sodium channel inactivation typically leads to a prolongation of the action potential in analogy to long-QT syndrome 3 [14]. In addition, a tendency towards prolongation of the ERP or PRR could be expected as an effect of sodium channel blockade.

One might not expect that, due to the enhancement of sodium channel inactivation, lacosamide would lead to a prolongation of cardiac repolarization duration (even in the presence of QT prolonging drugs). The concept of repolarization reserve [26] provides a possible explanation in this regard. In this concept, the reduction of a repolarizing current or the persistence of a depolarizing sodium current does not immediately lead to action potential prolongation, as the reserve of repolarizing currents is not saturated. Only a further reduction of the repolarization capacities leads to an action potential prolongation or proarrhythmia. In this case, the combination of the I_Kr_-inhibiting sotalol with lacosamide led to a significant APD_90_ and QT prolongation in combination with a significantly increased arrhythmia incidence. One might speculate that lacosamide reduces the repolarization reserve, thereby potentiating sotalol’s repolarization-prolonging effects. Analogical observations were made in our working group [27]. This is also consistent with clinical observations that have not described significant QT prolongation with lacosamide [5].

An alternative explanation for the lacosamide-induced prolongation of action potential duration in the presence of sotalol could involve indirect effects; the inhibition of Na^+^ entry during the action potential phases 0–2 due to Na_V_ channel blockade by lacosamide might increase the transmembrane Na+ gradient. This could, in turn, enhance the activity of Na^+^/Ca^2+^ exchangers (NCX), which generate an electrogenic inward Na+ current, prolonging the plateau phase. This effect would be the opposite of that caused by inhibition of the late Na+ current component, which is primarily responsible for the repolarization duration shortening observed with lacosamide treatment alone.

Despite its impact on cardiac repolarization, lacosamide is associated with additional cardiac side effects. For example, lacosamide may induce atrial arrhythmias and conduction delays (such as atrioventricular block) [6,35]. Given these potential effects, lacosamide should be used with caution in patients who have other risk factors for arrhythmia, such as those taking medications that affect the cardiac conduction system, those with pre-existing cardiovascular conduction disorders, or patients with diabetic neuropathy [6]. Accordingly, a recent meta-analysis found that patients taking lacosamide are at an increased risk of arrhythmias [6].

### 3.4. Electrophysiological Effects of Lamotrigine

Perfusion with lamotrigine led to a significant decrease in APD_90_ but not in QT interval. A possible explanation for this divergent effect is QRS widening, which can explain a stable QT interval with reduced action potential duration. Such QRS widening is often due to conduction delays, which can occur under lamotrigine as a consequence of the sodium channel blockade described above [13]. To be more precise, lamotrigine inhibits both the peak and late sodium currents of NaV 1.5, exhibiting rapid kinetics and biophysical properties akin to those of the class Ib antiarrhythmic drug mexiletine [13].

A similar effect was observed after previous sotalol perfusion. Here, additive perfusion with lamotrigine did not cause a significant increase in APD_90_ but in QT interval, which could also be explained by QRS broadening at reduced conduction velocity. In line with this, additive perfusion with lamotrigine caused a significant prolongation of ERP. The significant arrhythmia suppressive effect of additive lamotrigine perfusion in the sotalol pretreated hearts is remarkable. The significantly prolonged PRR under lamotrigine perfusion provides an explanation for this. Our [36,37] and other research groups [38] were able to show that a significantly prolonged PRR can protect the myocardium from the occurrence of ventricular arrhythmias in the setting of pathologic QT prolongation. This is consistent with numerous observations, which were able to show that QT interval is not a reliable predictor of ventricular proarrhythmia [39,40], but rather dispersion of repolarization and refractory period or PRR are valuable predictors in this regard.

It is notable that lamotrigine has been associated with an increased risk of cardiac arrhythmias, particularly in patients with structural or conduction heart disorders [6,13]. However, the literature remains inconsistent. A recent Danish population-based cohort study of over 90,000 patients found no increased risk of all-cause mortality associated with lamotrigine use in patients with heart disease, nor did it identify an elevated risk of cardiac conduction disorders in individuals without cardiac comorbidities [41]. In contrast, a retrospective observational study using a large healthcare claims database of more than 160,000 patients reported an increased risk of ventricular tachycardia with lamotrigine compared to commonly prescribed alternatives [42]. In 2021, the US Food and Drug Administration released a safety warning regarding lamotrigine use in patients with heart disease, based on in vitro data suggesting that lamotrigine exhibits class Ib antiarrhythmic activity, potentially slowing ventricular conduction and promoting arrhythmias [43]. The proarrhythmic effects of drugs with class Ib properties, especially under pathological conditions, have been previously reported [44]. Furthermore, a recent large retrospective study reported a strong association between lamotrigine use and an increased risk of atrial fibrillation [45].

### 3.5. Limitations

Even though the Langendorff model of the isolated rabbit heart is an established model for the investigation of electrophysiological mechanisms [46], its applicability to the human heart remains limited. Therefore, further in vivo and clinical studies are needed to validate the findings and reinforce the conclusions of this study. Furthermore, our model does not allow direct cellular electrophysiology or the observation of transcriptional effects due to the temporal relationship between perfusion and recording. This study focuses on parameters of cardiac repolarization, although recent research has shown that lacosamide and lamotrigine may also have significant effects on cardiac depolarization. Further studies are needed to more comprehensively elucidate the impact of both drugs on cardiac depolarization, such as the PQ interval (in the presence of preserved AV conduction), QRS duration, and conduction velocity. In this study, cardiac biomarkers and inflammatory parameters were not assessed, which could have provided further insight into electrophysiological differences among individual hearts. Future studies are needed to better characterize the correlation between specific biomarkers (e.g., CK-MB, troponin, CRP) and electrophysiological alterations.

### 3.6. Pharmacokinetic Suitability

The concentrations of lacosamide and lamotrigine employed in this study are in accordance with the reported maximum plasma levels in humans following administration of both substances [47,48].

For lamotrigine, a therapeutic reference range of 3.0–14.0 mg/l has been suggested for the treatment of seizures [47], corresponding to approximately 11.7–54.7 µM. However, serum/plasma levels exceeding 14 mg/l are not uncommon, with over 15% of patients on lamotrigine therapy exhibiting higher concentrations [47]. In order to consider poor metabolizers and potential drug–drug interactions, a higher concentration of 100 µM was employed in this study.

The therapeutic reference range for lacosamide is 1–10 µg/mL [49], corresponding to approximately 4–40 µM. Therefore, the concentrations used in this study (10 µM and 50 µM) fall within the therapeutic and supratherapeutic range, respectively.

Furthermore, the concentrations are well below the LD50 according to the Safety Data Sheets according to the REACH Regulation (EC) 1907/2006 of 253 mg/kg for lacosamide and 205 mg/kg for lamotrigine.

## 4. Materials and Methods

The experimental protocol was authorized by the local animal care committee (Landesamt für Natur, Umwelt und Verbraucherschutz Nordrhein-Westfalen, Germany; file number: 81-02.05.50.21.004) and conducted in compliance with the Guide for the Care and Use of Laboratory Animals published by the US National Institutes of Health (NIH Publication No. 852-3, revised 1996) as well as the ARRIVE guidelines. In this study, no randomization was performed as each heart acted as its own control. The sample size was determined based on prior studies from our group with similar expected effect size. No animals were excluded from this study.

The entire experimental protocol was approved by the local laboratory animal science office and the local federal authority (Landesamt für Natur, Umwelt und Verbraucherschutz Nordrhein-Westfalen, file number: 81-02.05.50.21.004). In brief, a total of 67 rabbits were euthanized using thiopental, and the hearts were then prepared for Langendorff perfusion. Here, perfusion was performed retrogradely via the aorta.

Temperature- (38 °C) and pressure-controlled perfusion was performed with modified Krebs–Henseleit buffer (NaCl 118 mM, NaHCO_3_ 24.88 mM, D-glucose 5.55 mM, KCl 4.70 mM, Na-pyruvate 2 mM, CaCl_2_ 1.80 mM, KH_2_PO_4_ 1.18 mM, MgSO_4_ 0.83 mM).

Seven monophasic action potential catheters were placed epicardially on the heart, and one endocardial catheter was placed in the left ventricle. The electrodes used in this study were specifically designed and manufactured by the electromechanical workshops of our university hospital. Furthermore, a pseudo-12 lead ECG was recorded from the warming bath surrounding the heart. Mechanical AV nodal ablation was performed. Thereafter, the pacing protocol was started.

**Figure 5:**
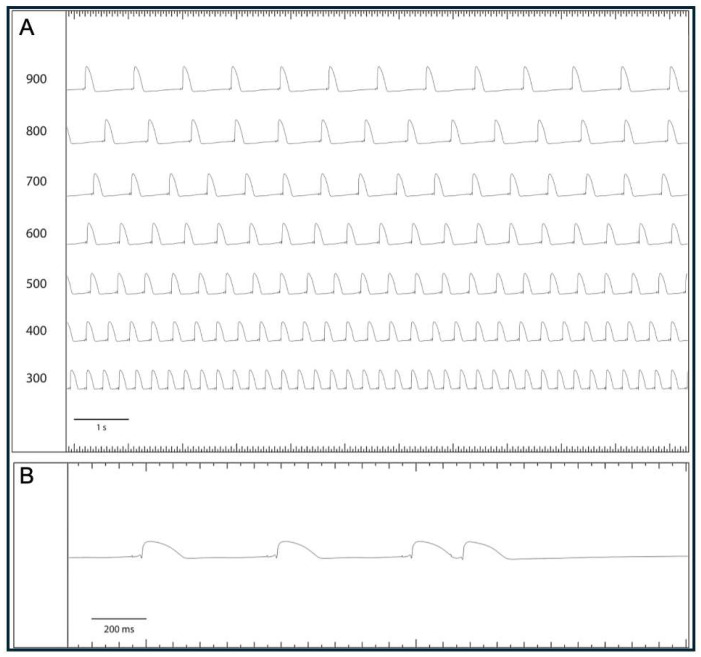
(**A**) Exemplary episode of monophysic action potential (MAP) recordings under decreasing stimulation frequency from 900 to 300 ms and baseline conditions. (**B**) Illustration of effective refractory period determination by S2 extrastimuli (MAP = monophasic action potential).

**Figure 6:**
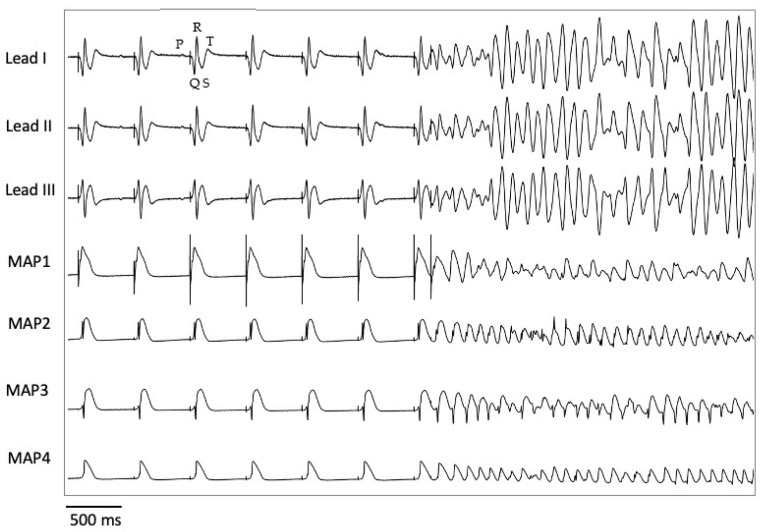
Induction of ventricular fibrillation by delivering a premature extrastimulus (S2) (MAP = monophasic action potential). The P wave, QRS complex, and T wave are represented by the corresponding deflections P, QRS, and T.

**Figure 7:**
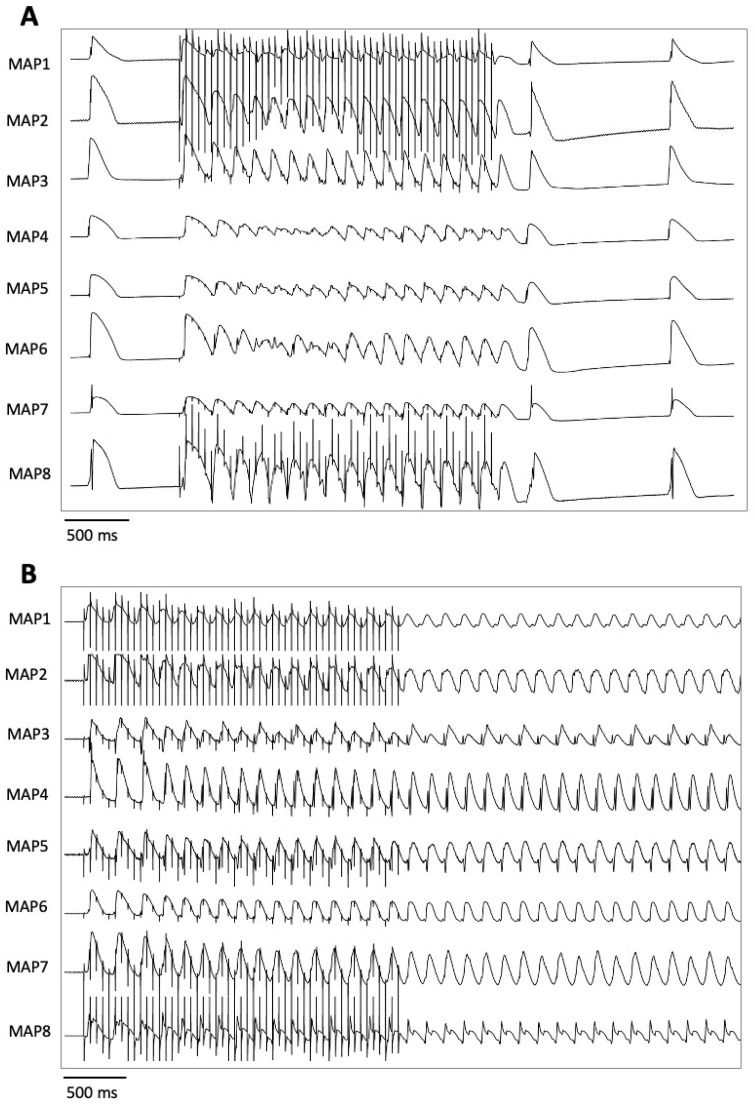
Illustrative example of a burst pacing without (**A**) and with (**B**) induction of a ventricular arrhythmia (MAP = monophasic action potential).

Pacing with seven different cycle lengths between 900 ms and 300 ms was performed, during which the action potential duration to 90% repolarization (APD_90_) and the QT interval were determined (Figure 5). Subsequently, pacing with a short-coupled extrastimulus was performed to determine the effective refractory period (ERP; Figure 6). In addition, repetitive burst stimulations (Figure 7) were used to record ventricular vulnerability. This was followed by perfusion with hypokalemic KHB (K^+^ 1.5 mM) to determine arrhythmia susceptibility in a hypokalemic environment. Spatial dispersion of repolarization was determined by the difference between the maximum and the minimum APD_90_ of the eight simultaneously recorded monophasic action potentials. Post-repolarization refractoriness (PRR) was calculated as the difference between ERP and APD_90_.

After the electrophysiological parameters were determined under baseline conditions, the allocation into four different perfusion groups was performed. In groups 1 and 3, lacosamide and lamotrigine were infused, respectively, following the collection of baseline data. Electrophysiological parameters and arrhythmia susceptibility were assessed at each concentration to determine concentration-dependent electrophysiologic effects. In groups 2 and 4, 100 µM sotalol was administered to reduce the repolarization reserve, provoke arrhythmias, and mimic a model of long-QT syndrome type 2. After repeating the protocol with sotalol, lacosamide or lamotrigine was subsequently added on top of sotalol in groups 2 and 4, respectively. To summarize, in group 1, 14 hearts (n = 14) were perfused with 10 µM lacosamide followed by 50 µM lacosamide. Group 2 (n = 15) was perfused with 100 µM sotalol followed by 50 µM lacosamide. Group 3 (n = 13) was perfused with 10 µM lamotrigine followed by 50 µM and 100 µM lamotrigine. Group 4 (n = 25) was perfused with 100 µM sotalol followed by 100 µM lamotrigine. The number of hearts per group was based on the number of trials in previous experiments and the individual signal quality per trial. The concentrations were selected based on the concentrations described in the literature in relation to observed plasma levels and single-cell studies on ion channel inhibition [12,13,47,48]. Electrograms and action potentials were recorded on a multi-channel recorder and digitized at a rate of 1 kHz with a 12-bit resolution. Variables are shown as mean ± standard deviation. Statistical analyses and graphic visualizations were performed employing Graphpad Prism Version 10. Drug effects on APD_90_, QT interval, spatial dispersion of repolarization, and effective refractory periods were analyzed using a mixed-effects model. Due to the partially missing measured values at different cycle lengths, a variance analysis in the form of an ANOVA (analysis of variance) with repeated measures was not suitable for the evaluation [50]. The exclusion of further measured values caused by the ANOVA would further reduce the data set and thus also significantly influence the statistical evaluation. Instead, the “mixed model” from the program GraphPad Prism (Version 10) was used as an approximation. This model uses a composite symmetric covariance matrix, taking into account “Restricted Maximum Likelihood”. In a data set without missing values, this model behaves very similarly to an ANOVA with measurement reproduction. However, in the case of missing values, it prevents the loss of the entire measurement series of the variable in question. The Geisser–Greenhouse correction was used in the statistical testing. The Tukey method was used to reduce the accumulation of alpha errors [51].

## 5. Conclusions

In the present study, lacosamide and lamotrigine showed divergent electrophysiological effects in a sensitive model of proarrhythmia. Under baseline conditions, both drugs led to a trend towards a decrease in repolarization duration without inducing substantial proarrhythmia. In a drug-induced model of long-QT syndrome type 2, lacosamide further prolonged cardiac repolarization duration, thereby amplifying the proarrhythmic risk. In contrast, lamotrigine suppressed arrhythmias by prolonging the post-repolarization refractoriness.

These findings suggest that careful consideration is advised when co-administering lacosamide with other drugs that prolong the QT interval, while lamotrigine appears safe even in combination with I_Kr_-blocking agents.

## Data Availability

The original contributions presented in this study are included in the article. Particularly due to their presentation as scatter plots, all raw data are included in the article. Further inquiries can be directed to the corresponding author.

## Abbreviations

The following abbreviations are used in this manuscript:

APD_90_	Action potential duration at 90% repolarization
ARRIVE	Animals in Research: Reporting In Vivo Experiments
ECG	Electrocardiogram
ERP	Effective refractory period
FDA	Food and Drug Administration
ICH	International Conference on Harmonisation of Technical Requirements for Registration of Pharmaceuticals for Human Use
IFM	Ile-Phe-Met
I_Kr_	Rapid component of the delayed rectifier potassium current
I_Na,L_	Late sodium current
KHB	Krebs–Henseleit buffer
LQTS	Long-QT syndrome
MAP	Monophasic action potential
Na_V_	Voltage-gated sodium channel
NCX	Na^+^/Ca^2+^ exchanger
NIH	National Institutes of Health
PRR	Post-repolarization refractoriness
SUDEP	Sudden unexpected death in epilepsy
VF	Ventricular fibrillation
VT	Ventricular tachycardia
